# The emotion regulation motive of nonsuicidal self-injury mediates the relationship between motor impulsivity and NSSI frequency in adolescents

**DOI:** 10.3389/fpsyt.2025.1692721

**Published:** 2025-11-21

**Authors:** Hong Ma, Xiaoli Liu, Xiangju Du, Danni Chi, Yuanyuan Zhang, Haihang Yu, Dongsheng Zhou

**Affiliations:** Department of Psychiatry, Affiliated Kangning Hospital of Ningbo University (Ningbo Kangning Hospital), Ningbo, Zhejiang, China

**Keywords:** nonsuicidal self-injury, motor impulsivity, impulsivity, emotion regulation motive, adolescents

## Abstract

**Background:**

Nonsuicidal self-injury (NSSI) is a common and acute mental health issue among hospitalised adolescents. Although prior research has highlighted the roles of both impulsivity and emotion regulation in self-injurious behaviours, the specific mediating role of the emotion regulation motive in the relationship between motor impulsivity and NSSI frequency remains insufficiently understood.

**Methods:**

206 adolescents with a history of NSSI were recruited from the Affiliated Kangning Hospital of Ningbo University. Subjects filled out the Ottawa Self-Injury Inventory (OSI) to evaluate the frequency and motives of NSSI behaviours, and the Barratt Impulsivity Scale-11 (BIS-11) to assess impulsivity. We conducted a mediation analysis and employed Causal mediation analysis to test whether emotional regulation function mediates the relationship between motor impulsivity and NSSI frequency.

**Results:**

The results showed positive correlations between motor impulsivity and frequency of NSSI (*r* = 0.21, *p* < 0.01) and emotion regulation motive (*r* = 0.34, *p* < 0.01). Causal mediation analysis revealed that motor impulsivity significantly influenced NSSI frequency through emotion regulation, with no direct effect observed (all ADEs, p > 0.05). Specifically, higher motor impulsivity was linked to increased probabilities of engaging in weekly (ACME = 0.0030, p < 0.001) and daily NSSI (ACME = 0.0017, p < 0.001), while emotion regulation mediated approximately 80% of the total effect.

**Conclusion:**

The study demonstrates that higher motor impulsivity is associated with a greater likelihood of engaging in weekly and daily NSSI, with emotion regulation motive significantly mediating this relationship. This highlights the need for interventions targeting impulsivity and emotion regulation to address NSSI behaviours in this population effectively.

## Introduction

Nonsuicidal self-injury (NSSI) is common among adolescents, especially in psychiatric inpatient settings, where its prevalence can exceed 60% (1, 2). In these environments, NSSI often reflects significant psychological distress, including emotional dysregulation, impaired functioning, and comorbid conditions (3). Adolescents in inpatient care are particularly vulnerable due to acute stress, emotional reactivity, and limited coping strategies, making them at high risk for future suicidal behaviour (4, 5). Understanding the mechanisms behind NSSI in this population is essential to address immediate risks and prevent long-term issues, such as chronic emotional dysregulation and treatment resistance (6). NSSI in hospitalised adolescents serves as a critical marker of broader psychopathological vulnerabilities, highlighting the need for targeted interventions (7).

Many scholars view NSSI as a behaviour that serves various intra- and interpersonal functions (e.g., emotion regulation, social influence, sensation seeking) (8, 9). Accordingly, emotion regulation thus seems to be the most common and clinically relevant construct, especially for adolescents who use such behaviour to self-soothe after feeling great emotional pain and psychological distress (10, 11). Recent studies have found a high degree of emotion regulation deficits associated with the frequency and severity of self-injury in adolescents who self-injure (12, 13). These results highlight the importance of studying emotion regulation processes in the understanding and treatment of NSSI, particularly among inpatient adolescents, who are especially at risk for intense emotional dysregulation and discomfort (14). For these relationships, emotion regulation has emerged as a primary target in the search for effective interventions for at-risk adolescents engaging in NSSI.

Although emotional dysregulation is associated with NSSI, motor impulsivity—the challenge of stopping automatic motor responses—is among the strongest predictors of NSSI (2, 15). Neuroimaging evidence demonstrates that deficits in motor inhibition most strongly correlate with NSSI frequency, mediating the relationship between increased frontoparietal white matter volume and self-injurious behaviours in clinical samples (16). Additionally, performance on task-based paradigms such as the Go/No-Go and Stop-Signal tasks supports a significant association between motor impulsivity and both NSSI and suicidal behaviours in adolescents (17). A recent meta-analysis of longitudinal studies reports that heightened impulsivity elevates the risk of engaging in NSSI by approximately 9%, acting as both a trait-based vulnerability and an immediate precipitant, particularly in emotionally dysregulated contexts (16). Despite accumulating cross-sectional, neuroimaging, and longitudinal data, targeted investigations within inpatient adolescent populations are notably lacking. Given the heightened emotional dysregulation and acute stress inherent to hospitalisation (18), elucidating the distinct contribution of motor impulsivity and its interplay with emotion-regulation deficiencies is imperative for clarifying mechanisms driving NSSI initiation and escalation in this high-risk group.

Recent studies have demonstrated a complex relationship between impulsivity and emotion dysregulation, and individuals high on some dimensions of impulsivity may be particularly at risk of using self-injury as a maladaptive strategy for emotion regulation (19). High motor impulsivity was related to a lack of emotional regulation and high emotional reactivity in adolescents, increasing vulnerability to NSSI (20). This dynamic between motor impulsivity and emotion regulation was of significance in the explanation of NSSI but is particularly salient in inpatient adolescents, where severe emotional dysregulation and heightened distress were commonly observed (21).

**Figure 1:**
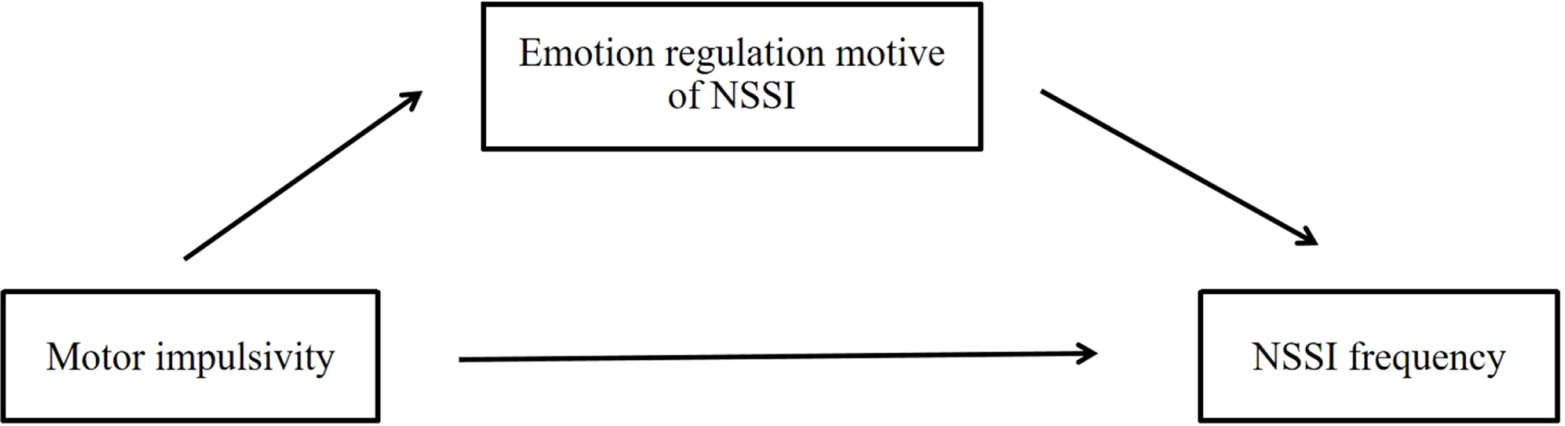
The hypothesis model diagram of the mediating effect.

Based on such widely used models of impulsive behaviour and emotion regulation, the present investigation will examine whether motor impulsivity is an essential factor associated with NSSI, in part, via its impact on the emotion regulation motive of NSSI. More specifically, we propose a theoretical model where the emotional regulation motive of NSSI acts as a mediator between motor impulsivity and NSSI behaviours (**Figure 1**). This model raises a critical empirical question: Can emotion regulation motive help explain how impulsive tendencies contribute to increased self-injury in clinical populations? To address this question, we tested two hypotheses: (1) motor impulsivity positively correlates with NSSI frequency, and (2) the emotion regulation motive of NSSI mediates this association. By examining this model in a sample of hospitalised adolescents, this study aims to provide novel insights into the psychological mechanisms driving NSSI, potentially guiding more targeted and effective clinical interventions for this vulnerable group.

## Methods

### Participants

From April 2021 to December 2023, 238 adolescents meeting criteria for NSSI were consecutively recruited from the Affiliated Kangning Hospital of Ningbo University. Written informed consent was obtained from all participants and their legal guardians before enrollment. Participants between 12 and 19 years old who met the diagnostic criteria for NSSI disorder outlined in the DSM-5 were included. Specifically, Criterion A—defined as engaging in NSSI on five or more separate days within the preceding 12 months—was assessed using a validated, structured diagnostic interview adapted into Chinese. Exclusion criteria included (1) the presence of behavioural disturbances severe enough to impede completion of assessments or failure to complete the study protocol, (2) fewer than five self-injurious episodes in the past year, and (3) a recent history of suicide attempts, current suicidal ideation with a specific plan, or acute suicide risk. Participant eligibility was determined through independent evaluations by two senior psychiatrists. This study adhered to the ethical principles of the Declaration of Helsinki and was approved by the Institutional Review Board of the Affiliated Kangning Hospital of Ningbo University (Approval No. NBKNYY-2021-LC-36).

### Procedure

Upon admission, participants were asked to complete standardised self-report questionnaires evaluating demographic characteristics (e.g., age, gender) and clinical variables, including NSSI frequency. Data collection commenced no earlier than the second day of hospitalisation to ensure participants were in a stable psychological state and to enhance the reliability of self-reported information. Under the supervision of trained research personnel, participants completed the Ottawa Self-Injury Inventory (OSI) and the Barratt Impulsiveness Scale–11 (BIS-11). Adolescents discharged within 24 hours of admission or admitted solely for brief observation were excluded from the analysis.

### Measures

#### Ottawa Self-Injury Inventory

The OSI (22) is a comprehensive, multidimensional self-report instrument that assesses various NSSI characteristics, including initiation, frequency, motivation to cease, typology, and functional motives. Participants rated the relevance of 31 NSSI motivation items (e.g., “to relieve unbearable tension”) on a 5-point Likert scale (0 = never a reason to 4 = always a reason). Recent NSSI frequency (past month) was assessed with the question, “How often in the past month have you hurt yourself without intending to die?” Responses were categorised as 0 = not at all, 1 = at least once, 2 = weekly, and 3 = daily. The OSI has demonstrated strong psychometric properties in clinical and non-clinical samples, with internal consistency coefficients ranging from 0.67 to 0.87.

#### Barratt Impulsiveness Scale-11

The BIS-11 (23) is a 30-item instrument that evaluates trait impulsivity across three subdomains: non-planning, motor, and cognitive impulsiveness. Each item is rated on a 5-point Likert scale (1 = never to 5 = very often), with higher scores indicating elevated levels of impulsivity. The Chinese adaptation of the BIS-11 has shown sound reliability and construct validity in adolescent cohorts. In the present sample, the internal consistency for the total score was excellent, with a Cronbach’s alpha coefficient of 0.916.

### Statistical analyses

Data were screened for missing values, outliers, linearity, and multicollinearity before analysis; no significant violations of assumptions were observed. All statistical analyses were performed using IBM SPSS Statistics version 25.0 and R software version 4.1.3. A two-tailed significance level of p < 0.05 was adopted throughout. Principal Component Analysis (PCA): PCA with direct oblimin rotation was used to identify potential dimensions of NSSI motives. Specifically, PCA was performed to confirm the factor structure of the Ottawa Self-Injury Inventory (OSI) motivation items before these identified factors were used in the mediation analysis. This step was crucial to ensure that the factors derived from the OSI’s motivation items were valid, reliable, and theoretically meaningful. The resulting factors were then employed as independent variables in the subsequent mediation model to explore their relationships with other key variables. Kendall’s tau-b correlation: This was used to explore the relationships among key variables. Causal Mediation Analysis: Mediation effects were tested using the causal mediation model in R software version 4.1.3. Causal mediation analysis was conducted to decompose the total effect of outcomes into direct and indirect effects, with specific variables mediating the indirect effect. The analysis included the average causal mediation effect (ACME), average direct effect (ADE), and the proportion of mediation, using 1,000 bias-corrected bootstrap samples to estimate path coefficients and corresponding p-values. Statistical significance of indirect effects was determined by 95% confidence intervals (CIs); mediation was considered significant if the CI did not include zero.

## Results

### Descriptive statistics

**Figure 2:**
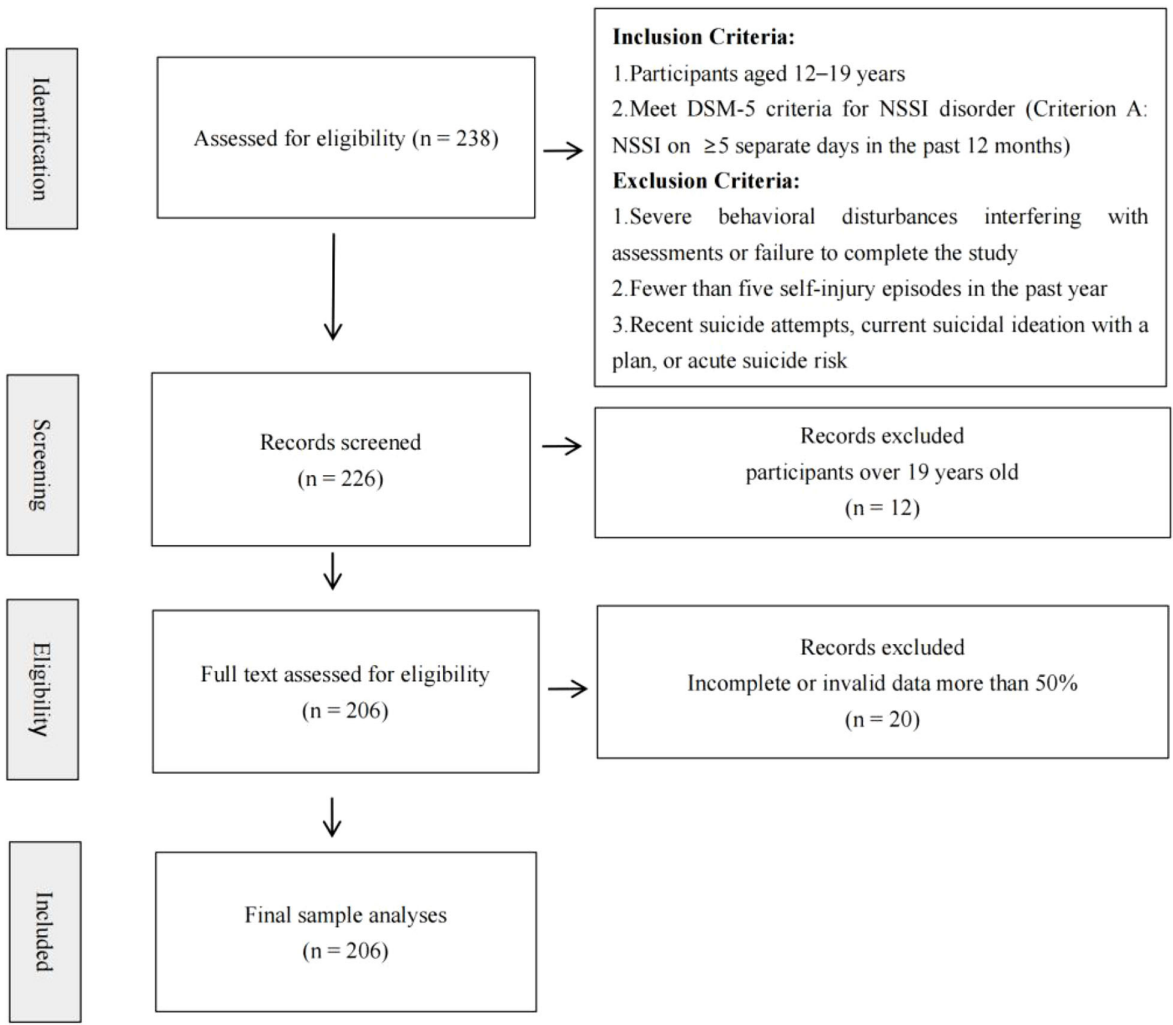
Study selection flow diagram.

During the recruitment period, 238 adolescents participated in the study. After excluding 20 participants with more than 50% missing data and 12 participants over 19, the final sample comprised 206 adolescents (Mage = 14.73, SD age = 1.74). The Consort Flow Diagram is shown in **Figure 2**.16.02% of participants were male, and 83.98% were female. Among the various mental disorders, depressive disorders had the highest prevalence (n = 153, 74.27%), followed by childhood emotional disorders (n = 17, 8.25%), anxiety disorders (n = 13, 6.31%), impulse control disorders (n = 2, 0.97%), and eating disorders (n = 2, 0.97%). NSSI frequency in the past month varied: 41.26% (n = 85) of participants reported daily self-injury, 26.70% (n = 55) reported weekly self-injury, 24.76% (n = 51) reported self-injury at least once, and 7.28% (n = 15) reported no self-injury.

### PCA: Principal component analysis of functional dimensions

PCA with direct oblimin rotation revealed a three-factor solution underlying the functions of NSSI, accounting for 47.72% of the total variance (see **Table 1**). The identified factors included emotion regulation function (items 1, 4, 8, 10, 13, 17, 21, and 26), social influence function (items 9, 11, 12, 14, 20, and 27), and sensation-seeking function (items 2, 7, and 29). Items that exhibited substantial cross-loadings or were part of factors with fewer than three items were omitted from the final structure to enhance factor clarity and ensure psychometric robustness. Internal consistency reliability analyses yielded Cronbach’s alpha coefficients of 0.904 for the Social Influence factor, 0.746 for Emotion Regulation, and 0.742 for Sensation Seeking. All coefficients exceeded the conventional threshold of 0.70, indicating satisfactory to excellent reliability across the extracted dimensions.

**Table 1:** Standardised Factor Loadings for the Three NSSI Motives Dimensions.

Motivations	Emotion regulation	Social influence	Sensation seeking
To release unbearable tension	0.839		
To stop feeling alone and empty	0.602		
To release feelings of tension or fears	0.782		
To distract me from unpleasant memories	0.761		
To release anger	0.788		
To help me escape from uncomfortable feelings or moods	0.763		
To relieve feelings of sadness or feeling “down”	0.794		
To release frustration	0.662		
To avoid getting into trouble for something I did		0.703	
To change my body image and/or appearance		0.696	
To belong to a group		0.637	
To stop my friends/boyfriend/or girlfriend from being angry with me		0.510	
To stop people from expecting so much from me		0.441	
To get out of doing something that I do not want to do		0.592	
To experience a”high” that feels like a drug high			0.822
To provide a sense of excitement that feels exhilarating			0.773
To prove to myself how much I can take			0.563
Cronbach’s α	0.904	0.746	0.742

*Factor loadings > 0.40 are presented. Items were retained if they loaded primarily on a single factor.

### Correlations for all study variables

**Table 2** summarises the results of the Kendall’s tau-b correlation analysis among the key study variables. Several noteworthy associations emerged. Motor impulsivity demonstrated a moderate and statistically significant positive correlation with NSSI frequency (*r* = 0.21,*p* < 0.01), as well as with two functional domains of NSSI, emotion regulation (*r* = 0.34, *p* < 0.01) and sensation seeking (*r* = 0.24, *p* < 0.01). The emotion regulation function showed moderate positive associations with NSSI frequency (*r* = 0.36, *p* < 0.01), social influence motives (*r* = 0.31, *p* < 0.01), and sensation seeking motives (*r* = 0.34, *p* < 0.01). Age was found to be negatively correlated with non-planning impulsivity (*r* = −0.14, *p* < 0.01) and cognitive impulsivity (*r* = −0.17, *p* < 0.01).

**Table 2:** Kendall’s tau-b correlation analysis of our study variables.

Variable	1	2	3	4	5	6	7	8	9
1. age	—								
2. sex	0.02	—							
3. No planning impulsivity	-0.14**	0.03	—						
4. Motor impulsivity	-0.04	0.04	0.33**	—					
5. Cognition impulsivity	-0.17**	0.03	0.61**	0.22**	—				
6. NSSI frequency	0.01	0.15*	0.06	0.21**	0.04	—			
7. Sensation seeking	0.05	0.10	0.04	0.17**	0.01	0.24**	—		
8. Emotion regulation	-0.03	0.08	0.12*	0.34**	0.10	0.36**	0.34**	—	
9. Social influence	-0.03	0.04	0.03	0.14**	0.04	0.16**	0.36**	0.31**	—

*N = 206. *p <.05, **p <.01 two-tailed. All variables were assessed using validated self-report instruments.

### Mediation analysis

**Figure 3:**
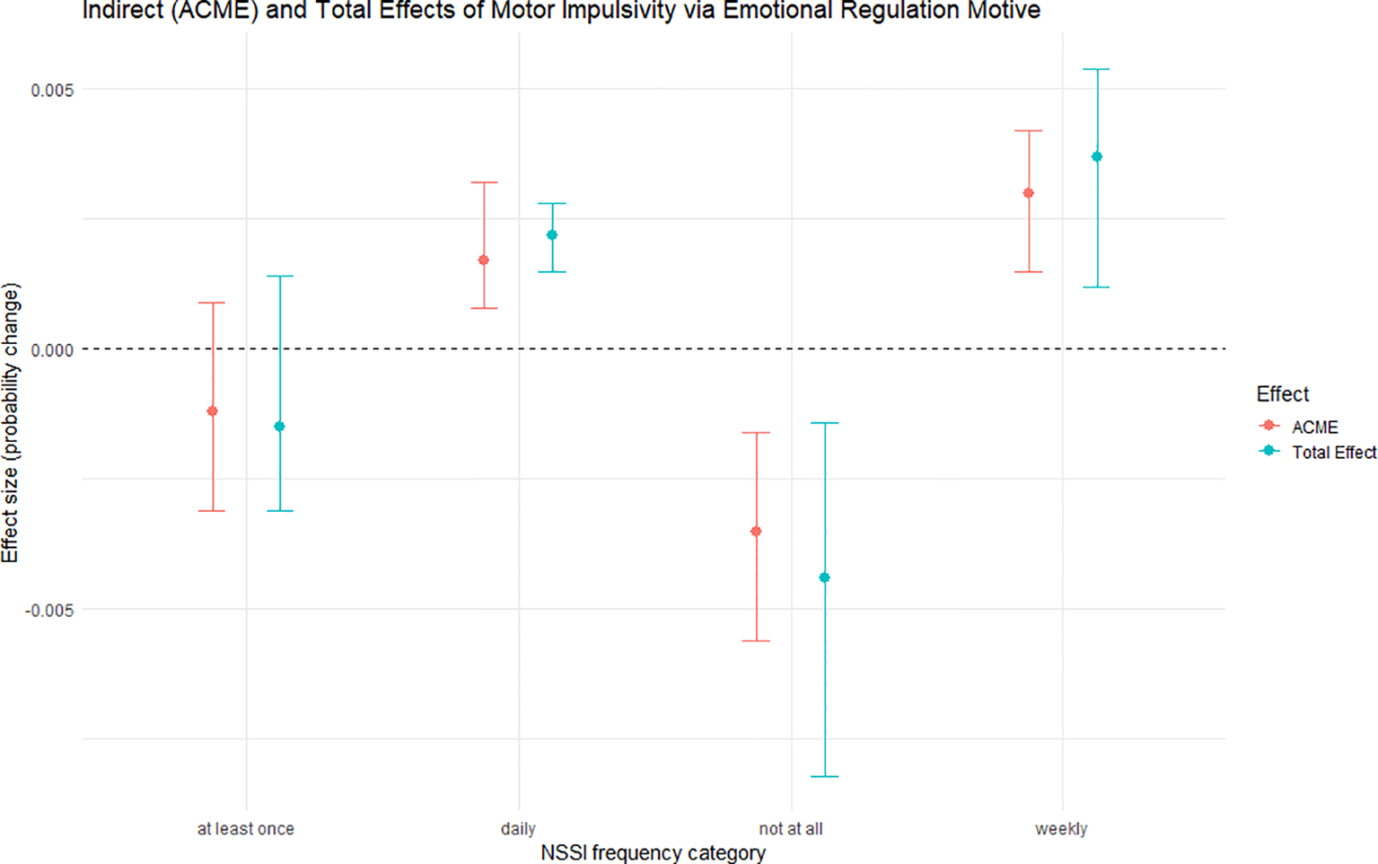
Indirect (ACME) and total effects of motor impulsivity on NSSI frequency categories via emotional regulation motive. Points indicate effect size estimates (probability change), with vertical bars representing 95% nonparametric bootstrap confidence intervals (percentile method, 1,000 resamples; N = 206). ACME = Average Causal Mediation Effect (indirect effect); Total Effect = combined indirect and direct impact. Negative values represent decreased probability of the corresponding NSSI category, and positive values represent increased probability.

As summarised in **Table 3** and illustrated in **Figure 3**, the causal mediation analysis revealed a significant indirect pathway through which motor impulsivity influenced the frequency of NSSI via the emotion regulation motive. Motor impulsivity was not directly associated with NSSI frequency (ADEs across outcome categories: *p* > 0.05). However, significant indirect effects through the emotion regulation motive were observed. Specifically, motor impulsivity was associated with a lower probability of being in the “Not at all” group (ACME = -0.0035, 95% CI [-0.0056, -0.0016], *p* < 0.001) and higher probabilities of being in the “weekly” (ACME = 0.0030, 95% CI [0.0015, 0.0042], *p* < 0.001) and “daily” (ACME = 0.0017, 95% CI [0.0008, 0.0032], *p* < 0.001) groups. No significant indirect effect was found for the “at least once” NSSI category (*p* > 0.05). The total effect mirrored the indirect effect pattern, indicating that the emotion regulation motive primarily accounted for the influence of motor impulsivity on NSSI frequency.

**Table 3:** Results of Causal mediation analysis for the effect of motor impulsivity on NSSI frequency via emotional regulation motive.

Effect type	Y=0	Y=1	Y=2	Y=3
ACME (indirect)	−0.00351***(95% CI [−0.00556, −0.00161])	−0.00122(95% CI [−0.00310, 0.00089])	0.00300***(95% CI [0.00151, 0.00418])	0.00173***(95% CI [0.00083, 0.00320])
ADE (direct)	−0.00087(95% CI [−0.00381, 0.00103])	−0.00029(95% CI [−0.00101, 0.00116])	0.00074(95% CI [−0.00084, 0.00248])	0.00042(95% CI [−0.00096, 0.00108])
Total Effect	−0.00436***(95% CI [−0.00822, −0.00143])	−0.00153(95% CI [−0.00309, 0.00140])	0.00373***(95% CI [0.00123, 0.00543])	0.00216***(95% CI [0.00150, 0.00283])
Proportion mediated	0.81 (≈80%)	—	0.80 (≈80%)	0.80 (≈80%)

(N = 206, ACME = Average Causal Mediation Effect; ADE = Average Direct Effect. Not at all: Y = 0, at least once: Y = 1, weekly: Y = 2, daily: Y = 3, 95% CI based on nonparametric bootstrap (percentile, 1000 resamples). p <.05 (*), <.01 (**), <.001 (***). Proportion mediated = ACME/Total Effect. Only significant proportions reported.

## Discussion

This study explored the association between motor impulsivity and NSSI frequency in hospitalised adolescents, focusing on the mediating role of emotion regulation motive. Our findings revealed that motor impulsivity was positively correlated with NSSI frequency, but the emotion regulation motive of NSSI entirely mediated this relationship. Adolescents exhibiting higher levels of motor impulsivity were more likely to engage in NSSI as a maladaptive strategy to manage negative emotional states. Notably, this mediating model accounted for approximately 80% of the total effect, highlighting the central role of emotion regulation in understanding NSSI in this population. These results suggest that while motor impulsivity serves as a risk factor for NSSI, its impact is primarily mediated by the adolescents’ reliance on NSSI as an emotional regulation motive.

We discovered that adolescents with higher motor impulsivity scores also reported greater frequencies of NSSI, in line with earlier reports that impulsivity was one of the strongest correlates of self-injury (24). Furthermore, our findings expand existing literature by demonstrating that the emotion regulation motive of NSSI mediated the effect of motor impulsivity on NSSI frequency. This suggests that impulsive adolescents are more susceptible to using self-injury as a means to alleviate emotional distress, in line with previous results of Vieira et al. (25) and Lockwood et al. (26). Importantly, the relationship between impulsivity and emotion regulation is illustrative of the complicated nature of NSSI behaviours, with highly impulsive adolescents likely to experience difficulty coping with emotions and thus are at greater risk of engaging in self-harm as a means of reducing emotional distress (27).

Impulsivity has been shown to interact with emotion regulation in predicting NSSI behaviour (28, 29). This study expands on these findings by modelling the emotion regulation motive of NSSI as a mediating factor in inpatient adolescents, a high-risk group for self-injury. Unlike previous research, which often treated these variables separately, we provide a more nuanced understanding by linking motor impulsivity to NSSI through emotion regulation. Our causal mediation analysis revealed that motor impulsivity did not directly affect NSSI frequency but significantly influenced it indirectly via emotion regulation. Specifically, motor impulsivity was associated with a lower likelihood of being in the “Not at all” NSSI group and higher probabilities of engaging in NSSI weekly or daily. These findings align with previous work by Midkiff et al. (30) and Andover & Morris (31), but our study highlights the importance of motor impulsivity in the pathway to NSSI. This integrated model enhances our understanding of NSSI and informs more targeted intervention strategies.

This research extends existing theoretical models of NSSI by combining the motor impulsivity and emotion regulation motive of NSSI into one framework. Our causal mediation analysis reveals that motor impulsivity indirectly influences NSSI frequency through emotion regulation, suggesting that impulsive traits are linked to NSSI behaviours by affecting emotional regulation strategies. This framework highlights the dynamic interaction between motor impulsivity and emotion regulation, providing insights into how impulsivity can manifest through emotional dysregulation and contribute to maladaptive coping strategies such as NSSI. Our findings suggest that the emotional regulation motive of NSSI, particularly among adolescents with high impulsivity, serves as the “bridge” that connects impulsive tendencies to the expression of self-injury (32). This model provides a potentially fruitful direction for future studies, especially in elucidating how impulsivity is related to other psychological processes (e.g., cognitive distortions, emotional sensitivity) in the aetiology and maintenance of NSSI (33, 34).

These findings have clinical implications for assessing and treating adolescents who engage in NSSI, especially those in an inpatient care program. Identifying emotion regulation motive as a core process mediates the relation between motor impulsivity and self-injurious behaviour has implications for treatment planning, including addressing the general inclination of impulsive responding and using maladaptive strategies for regulating emotions. Clinicians may want to add structured emotion regulation training (as in updated DBT) to impulse control interventions, especially for youth with high motor impulsivity (35, 36). Indeed, in clinical practice, the assessment of self-injury should not be limited to the frequency of the behaviour, but should also consider the motives and mechanisms underlying the self-injuring behaviour (37, 38). This methodology provides a better understanding of adolescent NSSI emotional relievers and offers personalised intervention opportunities (39). Attenuating this emotion-impulsivity pathway may be beneficial in enhancing treatment outcomes and lessening the recurrence of self-injury in high-risk youth (40).

Several limitations of this study should be noted. First, the cross-sectional design prevents causal inferences; longitudinal studies are needed to clarify the temporal relationships between motor impulsivity, emotion regulation, and NSSI frequency (40). Second, reliance on self-report data introduces potential biases, such as social desirability and recall errors. Future research could incorporate multi-method approaches, including behavioural measures and ecological momentary assessments, to improve data accuracy (41). Additionally, the sample was predominantly female (83.98%), limiting generalizability to males and non-inpatient populations. Excluding adolescents with recent suicidal ideation or attempts further restricts applicability to those at higher suicide risk. Comorbid conditions like depression, ADHD, or borderline personality traits were not accounted for, and medication use or hospitalisation length could also affect emotional regulation and impulsivity (3, 42).

Further research in the neurobiological correlates of the relationship between motor impulsivity and the emotion regulation function of NSSI against the background of NSSI behaviours is encouraged. Exploring the nature of those interactions in neural circuits may have translational implications for targeted intervention approaches (41, 43). Moreover, examining the functions of online platforms in emotion regulation and how they are connected to NSSI could identify novel modern coping strategies (44, 45). Cohort studies are required to assess physical activity’s causality and identify relatively early biomarkers in at-risk adolescents (46). In addition, the integration of machine learning could enhance the prediction model of NSSI and facilitate individualised intervention strategies (47, 48).

## Conclusion

Our findings revealed that higher motor impulsivity is associated with a greater likelihood of engaging in weekly and daily NSSI, with emotion regulation motive significantly mediating this relationship. This highlights the importance of targeting motor impulsiveness and emotion regulation skills in treatment. Further studies could also address the underlying neurobiology of these disorders and investigate the added value of digital technologies and machine learning to develop person-tailored strategies for prevention and early detection of NSSI.

## Data Availability

The raw data supporting the conclusions of this article will be made available by the authors, without undue reservation.
