# Supplementary material for: The emotion regulation motive of nonsuicidal self-injury mediates the relationship between motor impulsivity and NSSI frequency in adolescents
Source: Front Psychiatry. 2025 Nov 21;16:1692721. doi: 10.3389/fpsyt.2025.1692721 (PMC12678389; doi:10.3389/fpsyt.2025.1692721)
Supplement: Supplementary file 1 [file Table1.docx]

| Pattern Matrix**^a^** | | | | | | | |
| --- | --- | --- | --- | --- | --- | --- | --- |
|  | Component | | | | | | |
|  | 1 | 2 | 3 | 4 | 5 | 6 | 7 |
| 1.To release unbearable tension | .839 | .038 | .002 | -.047 | -.032 | -.077 | -.080 |
| 21.To relieve feelings of sadness or feeling “down” | .794 | -.145 | -.006 | .020 | -.042 | .162 | .076 |
| 13.To release anger | .788 | .154 | -.029 | -.054 | .031 | -.115 | .051 |
| 8.To release anger | .782 | -.030 | -.009 | .078 | .123 | .039 | -.023 |
| 17.To help me escape from uncomfortable feelings or moods | .763 | -.093 | .091 | .002 | .013 | .061 | .028 |
| 10.To distract me from unpleasant memories | .761 | -.006 | .137 | .046 | -.067 | .028 | -.001 |
| 26.To release frustration | .662 | .081 | .059 | .029 | .098 | .155 | .007 |
| 4.To stop feeling alone and empty | .602 | .119 | -.109 | -.008 | .203 | .116 | -.022 |
| 9.To avoid getting into trouble for something I did | .159 | .703 | .116 | -.193 | .156 | -.058 | -.041 |
| 11.To change my body image and/or appearance | .026 | .696 | -.158 | .198 | -.170 | .123 | .136 |
| 12.To belong to a group | -.067 | .637 | -.100 | .341 | -.056 | .000 | .060 |
| 27.To get out of doing something that I don’t want to do | .225 | .592 | .110 | -.129 | .215 | -.022 | -.169 |
| 14.To stop my friends/boyfriend/girlfriend from being angry with me | -.119 | .510 | -.098 | .297 | -.021 | .236 | .243 |
| 20.To stop people from expecting so much from me | .098 | .441 | .121 | -.122 | .125 | .267 | .021 |
| 23.To stop me from thinking about ideas of killing myself | .006 | -.067 | .978 | .059 | -.043 | .009 | .010 |
| 24.To stop me from acting out ideas of killing myself | .002 | -.065 | .974 | .038 | -.054 | .038 | .028 |
| 5.To get care or attention from other people | -.070 | .029 | .086 | .877 | .123 | -.019 | -.112 |
| 15.To show others how hurt or damaged I am | .103 | -.041 | .019 | .836 | .083 | -.025 | -.006 |
| 2.To experience a “high” that feels like a drug high | .143 | -.117 | -.045 | .052 | .822 | .020 | -.017 |
| 7.To provide a sense of excitement that feels exhilarating | .095 | -.043 | -.003 | .203 | .773 | .088 | -.005 |
| 29.To prove to myself how much I can take | -.070 | .225 | .041 | .015 | .563 | -.078 | .241 |
| 6.To punish myself | .091 | .073 | -.002 | -.056 | -.083 | .723 | .073 |
| 18.To satisfy voices inside or outside of me telling me to do it | .059 | .020 | .218 | -.065 | .098 | .488 | .193 |
| 25.To produce a sense of being real when I feel numb and “unreal” | .209 | .119 | .145 | .019 | .201 | .462 | -.100 |
| 19.To experience physical pain in one area, when the other pain I feel is unbearable | .402 | .065 | .137 | .141 | -.025 | .458 | -.105 |
| 28.For no reason that I know about—it just happens sometimes | .278 | -.144 | -.140 | .160 | .251 | .447 | .052 |
| 22.To have control in a situation where no one can influence me | -.025 | .302 | .235 | .080 | .276 | .412 | -.237 |
| 30.Others (please list) | -.007 | -.089 | .008 | -.126 | .096 | .174 | .858 |
| 16.To show others how strong or tough I am | -.110 | .370 | .129 | .065 | .279 | -.188 | .428 |
| 3.To stop my parents from being angry with me | .231 | .156 | .133 | .234 | -.138 | -.220 | .362 |
| Extraction Method: Principal Component Analysis.  Rotation Method: Kaiser Normalized Oblimin.^a^ | | | | | | | |
| a. The rotation converged after 15 iterations. | | | | | | | |
